# Evaluation of Micromovements and Stresses around Single Wide-Diameter and Double Implants for Replacing Mandibular Molar: A Three-Dimensional FEA

**DOI:** 10.5402/2012/680587

**Published:** 2012-03-07

**Authors:** Shrikar R. Desai, I. Karthikeyan, Rika Singh

**Affiliations:** Department of Periodontology & Implantology, H.K.E. Society's S. Nijalingappa Institute of Dental Sciences and Research, Gulbarga, PIN 585 105, India

## Abstract

*Purpose*. The purpose of this finite element study was to compare stresses, strains, and displacements of double versus single implant, in immediate loading for replacing mandibular molar. *Materials and Methods*. Two 3D FEM models were made to simulate implant designs. The first model used 6 mm wide-diameter implant to support a single molar crown. The second model used 3.75-3.75 double implant design. Each model was analyzed with a single force magnitude of 70 N in oblique axis in three locations. *Results*. This FEM study suggested that micromotion can be well controlled by both double implants and 6 mm single wide-diameter implant. The Von Mises stress for double implant had 31%–43% stress reduction compared to the 6 mm implant. *Conclusion*. Within the limitations of the paper, when the mesiodistal space for artificial tooth is more than 12.5 mm, under immediate loading, the double implant support should be considered.

## 1. Introduction

Threaded root form osseointegrated implants were designed originally to support complete arched fixed implant supported restorations for completely edentulous patients [1]. Now, this type of implant is used to support single-implant supported crowns and fixed partial dentures in partially edentulous areas [2]. Many in vitro and animal studies attempted to predict the biomechanical and clinical behavior of dental material and technique associated with implant-supported prosthesis [3–5]. In vitro methods include conventional in vitro model analyses [6], photo elastic analyses [4, 7, 8], and finite element analyses [9]. In vitro studies are less complicated and less expensive than clinical trials and produce results relatively quickly compared to randomized controlled trials [10]. FEM analysis has been used to provide analytical solutions to problems involving complex geometric forms [9].

Posterior teeth have two or three roots, having from 450 to 533 mm^2^ as a total anchorage area in good quality bone [11], whereas surface area of 3.75 mm implant varies from 72 to 256 mm^2^ depending on its length. The molar has a crown surface area of approximately 100 mm^2^, whereas 3.75 mm implant has cross section area 10.9 mm^2^. Therefore, the tooth can dissipate occlusal forces efficiently, whereas masticatory forces are exerted at angle mesiodistally and buccolingually creating bending and torquing vectors [12] on the implant. The cross-sectional area for 2 (3.75 mm) implants is 21.9 mm^2^, whereas for single (5 mm) implant 19.6 mm^2^ and for 2 (4 mm) implant 19.6 mm^2^ [13]. Greater dimension of bone buccolingually is required for placement of wide implant [14]. The wide implant is primarily a means of salvaging a procedure if a previous implant failed, a site had been over enlarged, or the operator desired to place an implant in a recent extraction socket [15, 16]. As a result, implants used as single molar replacements have high abutment fracture and loosening of screws. Placing of double implant more closely mimics the anatomy of roots being replaced and doubles the anchorage surface area; it also reduces the rotational forces, more technically demanding than the use of wide implants [13]. To reduce the risk of implant failure and increase the ability of posterior implant to tolerate occlusal forces [13], the option is to use double implants instead of a single wide-diameter implant.

To date, there are no studies comparing the single wide 6 mm implant with two 3.75 mm in an edentulous mandibular molar area with a mesiodistal width of >12.5 mm. The purpose of this finite element study was to compare displacements and stresses of double versus single wide implant for replacing mandibular molar.

## 2. Materials and Methods

**Figure 1:**
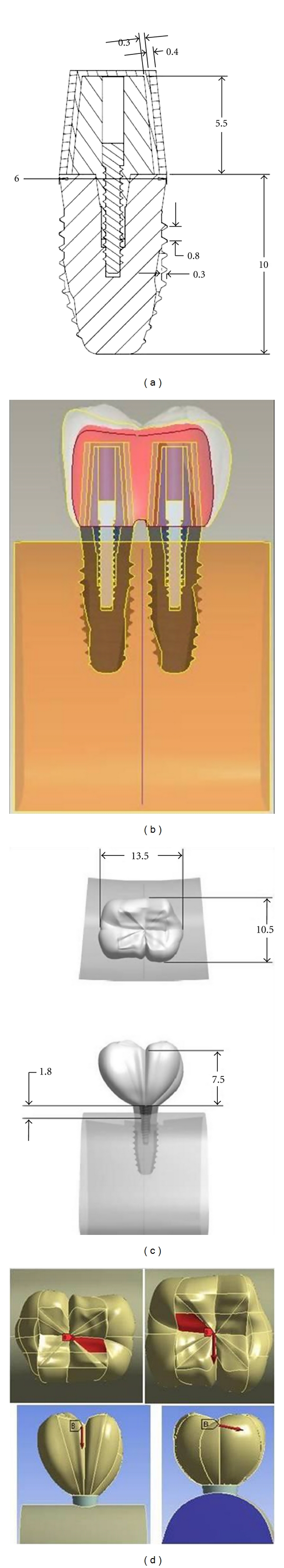
(a), (b), (c), and (d) show implant dimensions, crown dimensions, bone block, and loading condition.

Two 3D FEM models were made to simulate implant designs. The first model used 6 mm wide implant design consisted of 47614 nodes and 24920 elements. The second model used 3.75-3.75 double-implant design consisting of 65092 nodes and 33546 elements. (Figure 1(a)) The crown dimensions were derived from average dimensions of mandibular first molar [20]. (Figure 1(b)) 3D models were meshed using tetrahedral and octahedral elements and modeled by identifying the exact location of nodes after mathematical calculation by considering the inclination of threads. Each implant design consisted of fixture of 10 mm length incorporating V threads with a thickness of 0.2 mm and having a constant pitch length and height of 0.8 mm and 0.3 mm, respectively. Tapered implants with crestal diameters of 3.75 and 6 mm were used. Corresponding apical diameters were 2.4 and 4.1 mm, respectively. Abutment of height was 5.5 mm with a metal ceramic crown of dimensions 13.5 mm mesiodistally and 10.5 mm buccolingually, metal of thickness 0.4 mm, and a layer of cement between abutment and crown of thickness 0.3 mm. A smooth surface collar height of 1.8 mm was incorporated. The implant with the crown was placed in a bone block of height 18.5 mm and width 17.4 mm (Figure 1(c)). The bone consisted of 2 mm of cortical bone and the rest cancellous bone. Cortical and cancellous anisotropic properties were applied to the bone. The only difference between these two models was the number and diameter of implants. Each model was analyzed with 70 N with 15° to the vertical axis to produce a buccolingual direction of force (Figure 1(d)).

Loads were applied at 3 different locations [4]

1. the central fossa,
2. the buccolingual midpoint of the distal marginal ridge,
3. the distobuccal cusp tip.

The boundary conditions were defined by restraining all nodes at the base of 3D models. The modeling analyses were accomplished using a software program ANSYS work bench version 11. The material properties were derived from other studies [21–23] (Table 1). 

## 3. Results

For each implant design, the loading process 70 N on 3 locations generated displacements as follows.

**Central Fossa-70 N (15°) Oblique to Vertical Axes**

**Figure 2:**
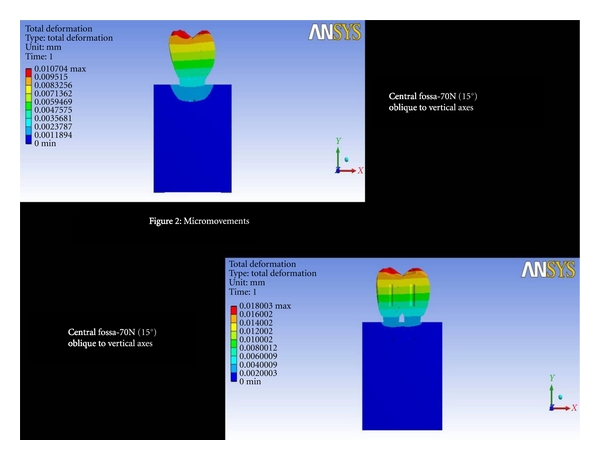
Total deformation at central fossa-70N (15°) oblique to vertical axes.

**Figure 3:**
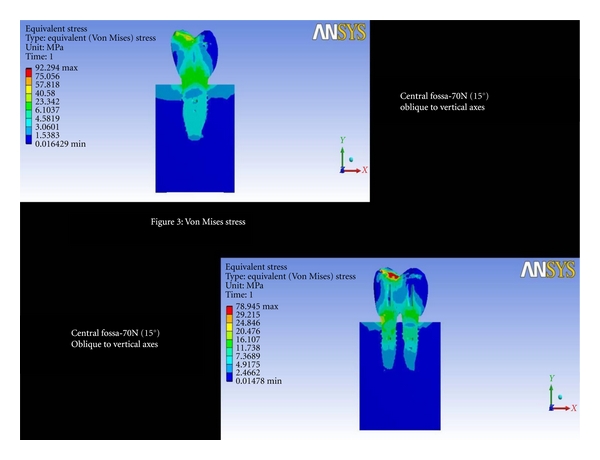
Von Mises stress at central fossa-70N (15°) oblique to vertical axes.

The total deformation was 0.004 mm for double implants and 0.00357 mm for 6 mm (Figure 2 and Figure 8). The Von Mises stress was 23.34 MPa for 6 mm wide implants, and the least value was recorded for double implants (16.10 MPa) (Figure 3 and Figure 9).

**Distal Marginal Ridge-70 (15°) Oblique to Vertical Axes**

**Figure 4:**
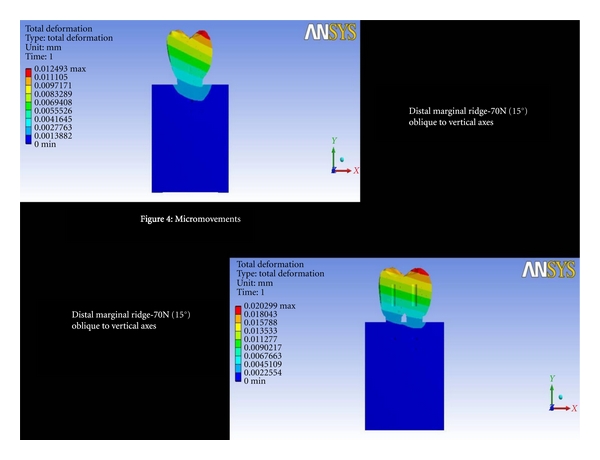
Total deformation at distal marginal ridge-70N (15°) oblique to vertical axes.

**Figure 5:**
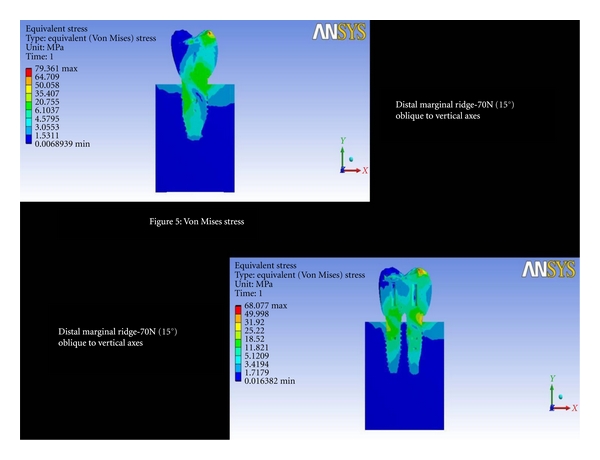
Von Mises stresses at distal marginal ridge-70N (15°) oblique to vertical axes.

The total deformation was 0.0045 mm for double implants and 0.0041 mm for 6 mm (Figure 4 and Figure 8). The Von Mises stress was 20.75 MPa and 18.52 MPa for 6 mm and double implants recorded, respectively (Figure 5 and Figure 9).

**Distobuccal Cusp-70 N (15°) Oblique to Vertical Axes**

**Figure 6:**
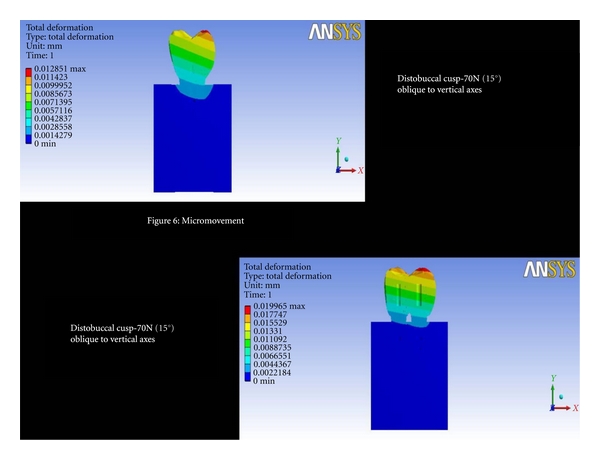
Total deformation at distobuccal cusp-70N (15°) oblique to vertical axes.

**Figure 7:**
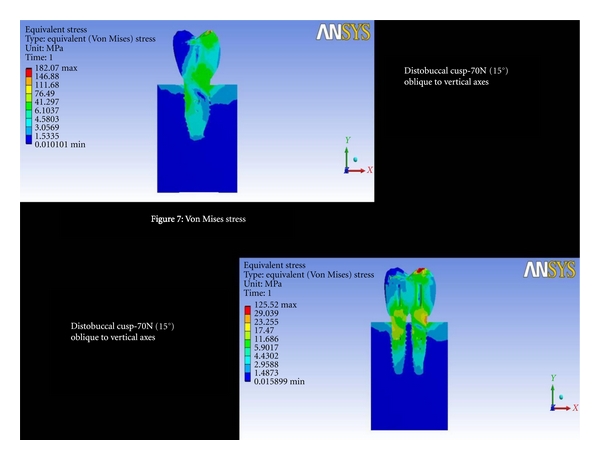
Von Mises stresses at distobuccal cusp-70N (15°) oblique to vertical axes.

**Figure 8:**
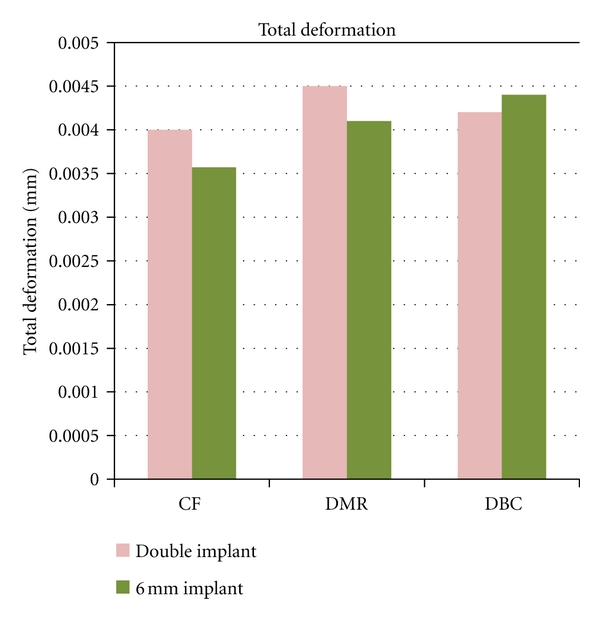
Graph showing total deformation.

**Figure 9:**
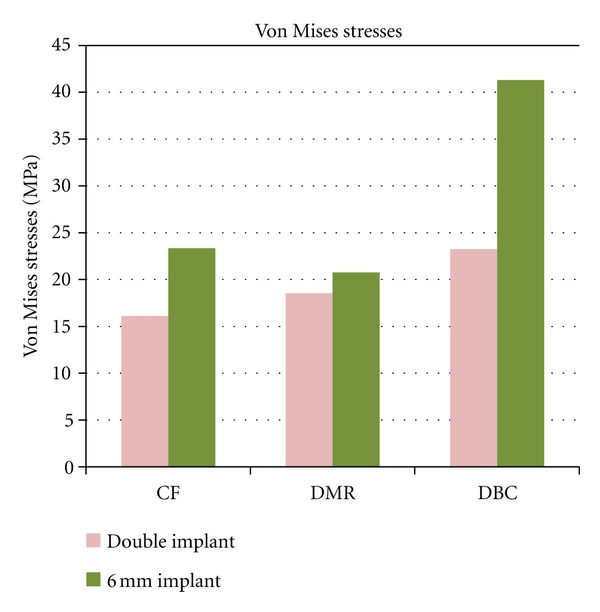
Graph showing Von Mises stresses.

The total deformation was 0.0042 mm for double implants and 0.0044 mm for 6 mm (Figure 6 and Figure 8). The Von Mises stress was 41.29 MPa for 6 mm compared to 23.25 MPa for double implants (Figure 7 and Figure 9).

## 4. Discussion

The present study was designed to compare wide-diameter and double implants for stresses, strains, and displacements for replacing mandibular molar. The present study design specifically addressed the problem of long span edentulous space of more than 12.5 mm. Finite element analysis is a numerical stress analysis technique that is widely used to study engineering and biomechanical problems [17, 18, 24]. 

Finite element analyses, a computer-based technique, calculates the behavior of engineering structures and their strength numerically. In the finite element method, a structure is broken down into many small simple blocks or elements. A simple set of equations describes the behavior of an individual element relatively. The structure will be build fully by joining together these set of elements, so the behavior of whole structure will be described by extremely large set of equations, which were actually the equations describing the behavior of individual elements joined together. The behavior of individual elements is assessed by computer from the solutions. Hence, the stress and deflection of all parts of the structure can be calculated [19]. 

The wide-diameter implants were used initially to replace standard diameter implants [14]. The introduction of wide implants for their high mechanical stability compared to standard (3.75 to 4 mm) diameter has led to its better success with excellent osseointegration due to increased surface area at the bone implant interface [25]. Despite encouraging data obtained from finite element analysis and animal studies, the initial experience with machined-surface wide-body implants showed lower success rates than those reported for standard-sized implants. Early clinical studies showed a failure rate ranging from 10% to 19% in the mandible and 9% to 29% in the maxilla [26, 27]. Furthermore, an augmented marginal bone resorption was observed around wide-body implants placed in the posterior mandible as compared to standard-sized implants [27]. Clinical reports have stated that wide implants tend to fail more frequently [28], and when the posterior edentulous ridges are narrow, the placement of wide implants will further lead to bone loss [29]. Placement of 6 mm wide-diameter implant would result in cantilevers of upto 5 mm on each marginal ridges of the crown in long span edentulous space of more than 12.5 mm. So the usage of this type of implants is limited due to aesthetic requirements for a natural emergence profile and width of the ridge.

The crown restored to one implant has certain discrepancy with its size to implants length and width. Cantilevers are generated when the size of the crown exceeds beyond implants long axis leading to screw loosening and eventually implant fatigue. The ideal replacement is with two implants for a single molar. According to Saadoun et al. [30], a minimum of 12.5 to 14.0 mm of interdental space is needed to successfully replace double-standard implants for a missing molar.

This study focused on the values of displacements and Von Mises stress on the surrounding bone [31]. The property of transverse isotropy was given to the cortical and cancellous bone and modeled as homogenous materials. A transversely isotropic material behaves identically in all planes perpendicular to the axis of symmetry. To relate stress to strain, transverse isotropy requires five independent elastic constants. The axis of symmetry for cortical bone is the mesiodistal axis of the jaw in transversely isotropic bone models, and it corresponds to the largest of young's modulus values for the cortical bone. The cancellous bone has superoinferior axis as axis of symmetry, and it is the smallest of young's modulus values for the same. The elements were 10-node tetrahedral structural solid p-elements (ANSYS solid 148) with three translational degrees of freedom at each node. Boundary conditions included constraining all three degrees of freedom at each of the nodes located at the most external mesial or distal aspect of the model [32]. It should be noted that great spectra of vertical loads/forces have been reported for patients with endosseous implants (mean range: 91–284 N), and the loads appear to be related to the location of the implant, as well as to food consistency. In finite element analysis, a combined load (oblique occlusal force) along with usual axial loads and horizontal forces (moment causing loads), as oblique force, gives local stresses in cortical bone [29], which is more realistic in directing occlusal forces than the others. Measured bond strengths of many base metal-porcelain combinations are comparable to those of noble alloy porcelain combinations [33]. Co-Cr alloys have high tensile strength (552 to 1034 Mpa) and high elastic modulus (200.000 Mpa). The Co-Cr alloy used in the present study was also used by Williams et al. [34]. These authors stated that Co-Cr alloy allowed more uniform distribution of stress within the framework, providing more efficient and durable load transfer. Porcelain is a commonly used material for occlusal surfaces [35]. Cibirka et al., in an in vitro simulated study, compared the force transmitted to human bone by gold, porcelain, and resin occlusal surfaces and found no significant differences in the force absorption quotient of the occlusal surfaces among these 3 materials. Therefore, porcelain was used for the occlusal surface [35].

The process of loosening failure in implants is one of the important determinant for the lack of primary stability [36]. Relative micromovements of about 100 or 200 *μ*m delivered by physiologic loads in bone implant interface may result in formation of a fibrous tissue layer inhibiting bone ingrowth, which then loosens the implant [37]. These relative micromovements in the bone implant interface need accurate evaluation, as they are of more concern in preclinical and clinical contexts [19]. There was negligible difference between micromovements of 6 mm and 3.75 mm double implants in all three locations on mandibular molar. This FEM study suggested that micromotion can be well controlled by double implants as well 6 mm single wide-diameter implant. Von Mises stress reduction was achieved better by 3.75-3.75 mm implant design compared to 6 mm, and the difference in percentage of stress reduction of 6 mm compared to double implant was from 31% to 43%. The concept of reducing implant-bone stress by means of two implants is a biomechanically more advantageous solution, not only for minimizing the mechanical problems such as screw loosening, but primarily for its all-over lower stress on implant and bone [13]. In case of deficient ridges, rather than aggressive protocols of augmentation procedures, if the mesiodistal width is >12.5 mm, the double implants can be placed with greater ease both for the patient and the operator.

Nevertheless, there were limitations of the study. The dynamic loads of chewing movements of the mandible were not applied and will have changes in stress patterns. Flexure of posterior mandible during opening and closing of mandible along with loads applied were not considered. The results of this study outweigh the limitations and give the clinician better options regarding varying diameter implants for replacing mandibular molar.

## 5. Summary and Conclusions

The present study compared four implant models, namely, 6 and 3.75-3.75 mm for replacement of mandibular molar using finite element method. Within the limitations of this FEM analysis, the following conclusions were drawn for immediate loading of mandibular molar replacing edentulous space of more than 12.5 mm.

- Von Mises stress reduction was achieved best by double implants compared to 6 mm implant.
- When the width of the ridge is adequate (8 mm) and the mesiodistal space is ≥12.5 mm, 6 mm implant could be used.
- When there is deficient ridge width (<8 mm) with mesiodistal space of ≥12.5 mm, double implants could be considered as they better control the stresses.
- The double implants give wider support to a molar restoration in both the mesial-distal and the buccolingual dimensions.

## Figures and Tables

**Table 1:** 

S no	Material	Youngs modulus (E MPa)	Poissons ratio (v)	Shear Modulus (GMPa)
(1)	Cortical bone [17]	E* x* 12,600	V* xy* 0.300 V* yz* 0.253	G* xy *4,850
		E* y* 12,600	V* xz* 0.253 V *yx* 0.300	G* yz* 5,700
		E *z* 19,400	V* zy* 0.390 V *zx* 0.390	G *xz* 5,700
(2)	Trabecular bone [17]	E* x* 1,148	V *xy* 0.055 V *yz* 0.010	G* xy* 68
		E *y* 210	V* xz* 0.322 V* yx* 0.010	G* yz* 68
		E* z* 1,148	V* zy* 0.055 V* zx* 0.322	G* xz* 434
(3)	Titanium [17]	110,000	0.350	
(4)	Porcelain [17]	70,000	0.190	
(5)	Cement [18]	12000	0.25	
(6)	Cobalt chromium metal [19]	87900	0.30	

Conflict of interest: Nil

Source of support: Nil.
